# SlSTOP1-regulated *SlHAK5* expression confers Al tolerance in tomato by facilitating citrate secretion from roots

**DOI:** 10.1093/hr/uhae282

**Published:** 2024-10-02

**Authors:** Huihui Zhu, Weiwei Chen, Zheng’an Yang, Congfang Zeng, Wei Fan, Jianli Yang

**Affiliations:** Key Laboratory of Vegetable Biology of Yunnan Province, College of Landscape and Horticulture, Yunnan Agricultural University, No. 452, Fengyuan Road, Panlong District, Kunming 650201, China; State Key Laboratory of Plant Physiology and Biochemistry, Institute of Plant Biology, College of Life Sciences, Zhejiang University, No. 866, Yuhangtang Road, Xihu District, Hangzhou 310058, China; College of Life and Environmental Sciences, Hangzhou Normal University, No. 2318, Yuhangtang Road, Xihu District, Hangzhou 311121, China; Key Laboratory of Vegetable Biology of Yunnan Province, College of Landscape and Horticulture, Yunnan Agricultural University, No. 452, Fengyuan Road, Panlong District, Kunming 650201, China; Agricultural and Rural Service Center, Huangguayuan Town, Yuanmou County 651308, Chuxiong Yi Autonomous Prefecture, China; Key Laboratory of Vegetable Biology of Yunnan Province, College of Landscape and Horticulture, Yunnan Agricultural University, No. 452, Fengyuan Road, Panlong District, Kunming 650201, China; Key Laboratory of Vegetable Biology of Yunnan Province, College of Landscape and Horticulture, Yunnan Agricultural University, No. 452, Fengyuan Road, Panlong District, Kunming 650201, China

## Abstract

SENSITIVE TO PROTON RHIZOTOXICITY 1 (STOP1) is a core transcription factor that regulates the expression of aluminum (Al) resistance genes to manage Al toxicity in plants. However, the genome-wide roles of SlSTOP1 in the Al stress response of tomato (*Solanum lycopersicum*) remain largely unknown. Here, we report that SlSTOP1 is crucial for Al tolerance in tomato, as loss-of-function mutants of *SlSTOP1* displayed hypersensitivity to Al stress. Aluminum stress had no effect on SlSTOP1 mRNA expression, but promoted accumulation of SlSTOP1 protein in the nucleus. Through integrated DNA affinity purification sequencing and RNA sequencing analysis, we identified 39 SlSTOP1-targeted Al-responsive genes, some of which are homologous to known Al resistance genes in other plant species, suggesting that these SlSTOP1-targeted genes play essential roles in Al resistance in tomato. Furthermore, using peak enrichment analysis of SlSTOP1-targeted sequences, we identified a *cis*-acting element bound by SlSTOP1 and validated this finding via dual-luciferase reporter and electrophoretic mobility shift assay (EMSA). Additionally, we demonstrated *SlHAK5* is one of direct targets of SlSTOP1 and functionally characterized it in terms of Al stress tolerance. Compared with wild-type plants, *Slhak5* mutants developed by CRISPR/Cas9 technology presented increased sensitivity to Al stress, which was associated with reduced citrate secretion from the roots. Together, our findings demonstrate that SlSTOP1 directly interacts with *cis*-acting elements located in the promoters of target genes involved in diverse pathways contributing to Al resistance in tomato.

## Introduction

Aluminum (Al) is widely found in the Earth’s crust as oxides and silicates, particularly in neutral and alkaline soils. However, when the soil pH decreases to <5.5, soluble cationic Al, specifically Al^3+^, is released into the soil, resulting in high toxicity to plant growth [1, 2]. The inhibition of root growth occurs within minutes of exposure to micromolar concentrations of Al, leading to impeded water and mineral nutrient absorption and 25%–80% yield losses [3, 4]. Therefore, Al toxicity has been recognized as a major challenge for crop production in acidic soils, which constitute ~40% of arable land worldwide [5].

To combat Al toxicity, plants have developed various strategies that can be classified as either external exclusion mechanisms or internal tolerance mechanisms [3]. External exclusion mechanisms prevent Al from entering root cells, whereas internal tolerance mechanisms involve complexing and compartmentalizing Al within cells. Among various external exclusion mechanisms, one extensively well-documented strategy is the Al-induced secretion of organic acid anions from the root apex, mainly malate, citrate, and oxalate, to chelate rhizospheric Al, rendering it harmless [2, 4]. Genes encoding transporters responsible for the Al-induced secretion of malate and citrate have been characterized as members of the *ALMT* (Al-activated malate transporter) and *MATE* (multidrug and toxic compound extrusion) families, respectively [2]. In addition to transporters capable of transporting organic anions, other transporters have also been demonstrated to be critical for A tolerance in plants. For example, the tonoplast-localized ATP-binding cassette (ABC) transporter ALS1 (for ALUMINUM SENSITIVE1) detoxifies intracellular Al by storing it within vacuoles [6]. In rice (*Oryza sativa*) and buckwheat (*Fagopyrum esculentum*), the vesicle-localized bacterial-type ABC transporters STAR1 (SENSITIVE TO Al RHIZOTOXICITY1) and STAR2 prevent extracellular Al binding by facilitating UDP-glucose-mediated modification of cell walls [7, 8].

AtSTOP1 (SENSITIVE TO PROTON RHIZOTOXICITY1) was initially identified by screening genes involved in low pH tolerance in Arabidopsis (*Arabidopsis thaliana*) [9]. Subsequent system biological and molecular genetic analyses revealed that AtSTOP1 regulates the expression of several known Al resistance genes, including *AtALMT1*, *AtMATE*, and *AtALS3*, to confer Al tolerance [10]. Interestingly, through promoter bioinformatics analyses, Tokizawa et al. demonstrated that AtSTOP1 directly binds to the promoters of *AtALMT1* and *AtGDH1 and AtGDH2* (*GLUTAMATE-DEHYDROGENASE1* AND *2*); however, weak binding to the promoter of *AtMATE* was observed, and no binding to the promoter of *AtALS3* was detected [11]. Therefore, the identification of STOP1-regulated targets is highly important for identifying novel genes and regulatory networks involved in Al tolerance. Moreover, notably, the role of AtSTOP1 and its orthologs appears to vary across plant species. For example, OsART1, a rice ortholog of AtSTOP1, regulates a distinct set of genes compared with those controlled by AtSTOP1 [12]. Similarly, VuSTOP1 from rice bean (*Vigna umbellata*) might regulate genes involved in proton tolerance rather than Al tolerance [13]. Therefore, characterizing AtSTOP1 orthologs and their direct targets from different plant species is crucial for obtaining a comprehensive understanding of Al tolerance mechanisms.

Tomato (*Solanum lycopersicum*) is one of most important commercial vegetables worldwide [14, 15]. However, tomato is relatively sensitive to Al toxicity among crops [16]; therefore, its productivity may be severely restricted when cultivated in acidic soils. For example, in Southwest China and South China, where acidic soils predominate, the tomato planting area is as high as 40 000 hm^2^. To date, our understanding of the physiological and molecular basis of Al toxicity and tolerance in tomato remains limited. Previous studies have demonstrated that Al stress can induce oxalate and citrate secretion in tomato roots as a mechanism to protect against Al toxicity [17]. Two genes, *SlAAE3*, encoding oxalyl-CoA synthetase, and *SlFDH*, encoding formate dehydrogenase, whose expression is regulated by SlNAC063 and SlSTOP1, respectively, have been reported to be involved in cytoplasmic oxalate degradation and Al tolerance regulation [15, 16]. Recently, the interaction between SlSZP1 (SlSTOP1-interacting zinc finger protein) and SlSTOP1 was shown to protect SlSTOP1 from SlRAE1-mediated degradation [18]. Nonetheless, our understanding of how tomato plants develop Al tolerance mechanisms remains largely unclear.

Here, we employed DNA affinity purification sequencing (DAP-seq) analysis, in conjunction with RNA-seq data, to identify Al-responsive genes directly targeted by SlSTOP1. A novel gene, *SlHAK5*, was found to be regulated by SlSTOP1, and functional analysis revealed that *SlHAK5* is involved in citrate secretion to confer Al tolerance in tomato. These findings provide novel insights into the STOP1-regulated Al resistance mechanism.

## Results

### SlSTOP1 positively regulates Al tolerance in tomato

To functionally characterize SlSTOP1 with respect to Al tolerance, we generated *ProSlSTOP1::GUS* and *35S::SlSTOP1::GFP* transgenic tomato reporter lines. GUS activity was observed throughout the entire root, flower, and fruit but was almost absent in the stems and leaves (Supplementary Fig. S1a, S1d). Similarly, we determined *SlSTOP1* gene expression in different organs and tissues at the flowering stage via real-time polymerase chain reaction (RT-PCR) and observed lower expression in stems and leaves (Supplementary Fig. S1c). Furthermore, Al treatment did not affect root GUS activity, indicating that *SlSTOP1* was constitutively expressed (Supplementary Fig. S1b). However, SlSTOP1-GFP accumulation was observed, as evidenced by the intensified GFP fluorescence under Al treatment (Supplementary Fig. S1e−f), which is consistent with the Al-induced nuclear accumulation of AtSTOP1 in *Arabidopsis* [19].

**Figure 1:**
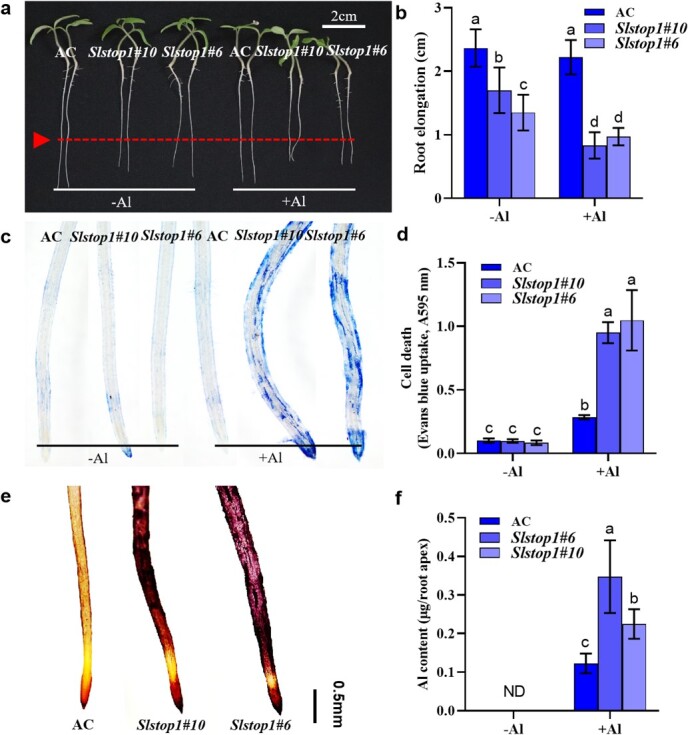
*Slstop1* mutants are hypersensitive to Al stress. (**a**–**b**) Phenotype of representative seedlings (**a**) and primary root elongation (**b**) of AC and two *Slstop1* mutants. The root elongation was measured before and after Al treatment. (**c**) Evans blue staining. After treatment, roots of AC and two *Slstop1* KO lines were stained with Evans blue staining solution, washed, and photographed by microscope. (**d**) The cell death by quantifying absorption of Evans Blue extract. Evans blue staining roots were extracted with 1% SDS and detected at A595 nm. (**e**) Hematoxylin staining of root Al content. Al-treated roots of AC and Slstop1 mutants were stained with hematoxylin, washed, and photographed. Bar = 0.5 mm. (**f**) The total Al content in 0- to 1-cm root tips of AC and two Slstop1 KO lines. After treatment, root apices were collected for extraction of Al and analyzed by ICP–MS. Seedlings of AC and *Slstop1* mutants with 3–4 cm length of primary root were treated with 0 or 5 μM Al for 48 h. Data are means ± SD. Different letters indicate significant differences among treatments by one-way ANOVA at the *P* ≤ 0.05 level. ND indicates not detected.

We previously developed SlSTOP1 loss-of-function mutants via CRISPR/Cas9 gene editing technology in the Micro-Tom background [16]. By using the same vector, we developed knockout (KO) lines of Ailsa Craig (AC) plants via *Agrobacterium tumefaciens*-mediated transformation. Two *SlSTOP1* KO lines (*Slstop1#10* and *Slstop1#6*) were chosen for further analysis. *Slstop1#10* contains a 148-bp deletion between the two targeted sites, and *Slstop1#6* contains a 48-bp deletion in the first targeted site and a 1-bp insertion in the second targeted site (Supplementary Fig. S2). We next compared the Al tolerance of the two *Slstop1* KO lines to that of the AC plants. After being subjected to 48 h of treatment with 5 μM Al, root elongation was significantly inhibited in the two *Slstop1* KO lines but not in the AC plants, suggesting that the *Slstop1* KO lines are hypersensitive to Al stress (Fig. 1a and b). Notably, the roots were shorter in the two *Slstop1* KO lines than in the AC plants in the absence of Al, indicating that SlSTOP1 is also crucial for low pH tolerance (pH 5.0 was used as a control). In agreement with greater root elongation inhibition in the two KO lines, more severe cell death was observed in the root tips of the *Slstop1* KO lines under Al treatment, as evidenced by Evans blue staining (Fig. 1c and d). Notably, obvious root swelling was observed for the two *Slstop1* KO lines, possibly resulting from the more severe inhibition of cell elongation, as has been reported in soybean (*Glycine max*) and rice [20, 21]. Furthermore, hematoxylin staining and inductively coupled plasma mass spectrometry (ICP–MS) analysis revealed greater Al contents in the root tips of both *Slstop1* lines than in those of the AC plants (Fig. 1e and f), suggesting that Al exclusion mechanisms are involved in SlSTOP1-regulated Al tolerance in tomato.

### Genome-wide identification of the direct targets of SlSTOP1

**Figure 2:**
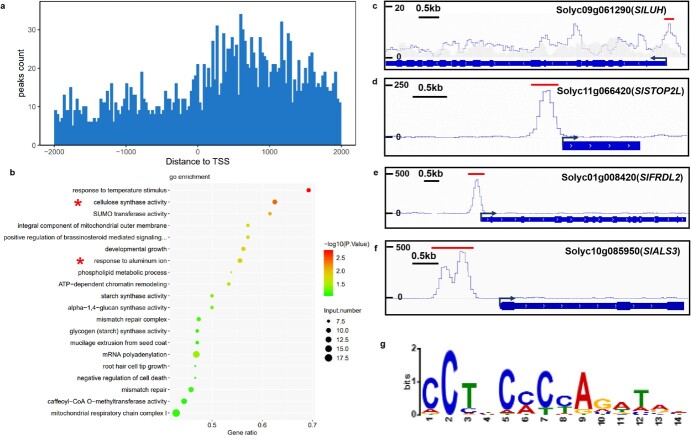
Genome-wide identification of SlSTOP1 targets by DAP-seq technology. (**a**) Genome-wide distribution of SlSTOP1 binding peaks near gene TSSs. (**b**) GO enrichment analysis for SlSTOP1 binding peak. Asterisk presents Al tolerance-related pathways. (**c–f**) Integrative Genome Browser displays SlSTOP1 binding efficiency and location of *SlLUH* (**c**), *SlSTOPL2* (**d**), *SlFRDL2* (**e**), and *SlALS3* (**f**) promoter. The height of peak represented the binding efficiency. The binding location was indicated by horizontal lines above the peaks. *SlLUH* location ranges from SL4.0ch09:54684472 to 54684701. *SlSTOP2L* location ranges from SL4.0ch11:50225601 to 50226115. *SlFRDL2* location ranges from SL4.0ch01:2451778 to 2452321. *SlALS3* location ranges from SL4.0ch10:64110426 to 64111247. (**g**) Enriched DNA motif identified in SlSTOP1 binding sites by MEME analysis.

Both the physiological and molecular mechanisms of Al tolerance have been well documented in *Arabidopsis* and rice; however, there is still a lack of understanding of these mechanisms in tomato. Since STOP1/ART1 is the master transcription factor that regulates genes involved in various physiological processes of Al tolerance, our interest lies in identifying the direct targets of SlSTOP1. For this purpose, we performed a DAP-seq assay to identify the binding targets of the SlSTOP1 protein genome-wide. Approximately 42–71 M clean reads were detected with more than a 99.8% mapping ratio, indicating the high quality of the DAP-seq results (Supplementary Table S1). A total of 7824 DAP-seq peaks overlapped between two biological replicates (Supplementary Table S2) and were distributed across different chromosomes (Supplementary Fig. S3a and b). Genome-wide distribution analysis revealed that these binding peaks were predominantly concentrated within 2-kb regions surrounding the gene transcriptional start sites (TSSs) (Fig. 2a), with up to 68% bound in distal intergenic regions (Supplementary Fig. S3c).

Gene Ontology (GO) enrichment analysis of all the peaks revealed several significantly enriched processes, including the process of ‘response to aluminum ions’, which was one of the top enriched terms (Fig. 2b). These findings suggest that SlSTOP1 plays pleiotropic roles in various aspects related to Al tolerance. For example, in the ‘cellulose synthase activity’ process, we observed a binding peak within the 1-kb promoter of *LUH* (*LEUNIG_HOMOLOG*) (Fig. 2c), whose homolog in Arabidopsis functions as a transcriptional co-repressor to modulate *AtPME46* (*PECTIN METHYLESTERASE46*) expression, thereby mediating Al sensitivity [22]. In the ‘response to aluminum ions’ process, *SlSTOP2L* (an ortholog of *AtSTOP1*), *SlFRDL2* (an ortholog of *AtMATE-*mediating citrate secretion [23]), and *SlALS3* (an ortholog of *AtALS3* responsible for Al redistribution [24]) were identified as the direct targets of SlSTOP1 (Fig. 2d−f).

To further explore the *cis*-acting elements of SlSTOP1, 703 peaks enriched in promoter regions were subjected to motif analysis via MEME [25]. Interestingly, we identified a binding motif for SlSTOP1 with a core sequence of 5′-CCTTCCCCAGATAA-3′, which strikingly resembles our previously reported SlSTOP1-binding motif in the promoter of *SlFDH* [16]. Therefore, the identified binding site is highly likely critical for interaction with SlSTOP1 and the consequent transcriptional regulation of target genes.

### Identification of Al-responsive genes directly regulated by SlSTOP1

To identify Al-responsive genes directly targeted by SlSTOP1, a comparative transcriptome analysis was performed for the root tips of the AC and *Slstop1* KO lines. First, to minimize the secondary effects of Al stress, we performed preliminary experiments to determine the optimal duration and concentration of Al treatment. Compared with that in the AC plants, the time course of the experiment revealed slight but significant inhibition of root elongation in the two *Slstop1* KO lines after being exposed to 5 μM Al for 6 h, followed by more pronounced inhibition with increasing treatment time (Supplementary Fig. S4). Furthermore, a dose-dependent experiment demonstrated that 5 μM Al was sufficient to significantly inhibit root elongation in both *Slstop1* mutants and AC plants (Supplementary Fig. S4b). Therefore, Al concentrations of 5 μM and 6 h of treatment were selected for transcriptomic analysis, under which the differentially expressed genes reflected, to the greatest extent, the true SlSTOP1-regulated genes directly related to Al tolerance.

Next, we conducted a comparative analysis of gene expression profiles through RNA-seq. The results revealed that exposure to Al led to a total of 1334 and 2049 significantly differentially expressed genes (DEGs) in the roots of AC and the *Slstop1* mutants, respectively (Supplementary Table S3–4). When the *Slstop1* mutants were compared with AC plants, we detected 192 DEGs without Al treatment and 1158 DEGs with Al treatment (Supplementary Table S5–6; Supplementary Fig. S5a). To identify SlSTOP1-regulated genes under Al treatment, we identified the overlapping DEGs among ‘Slstop1−Al vs. AC−Al’, ‘Slstop1+Al vs. AC+Al’, and Al-upregulated genes in AC plants (Supplementary Fig. S5b). A Venn diagram analysis revealed that a total of 327 upregulated DEGs were regulated by SlSTOP1. Among them, 82 DEGs were regulated by SlSTOP1 independent of Al, 34 DEGs were regulated by SlSTOP1 only in the absence of Al, and 211 DEGs were regulated by SlSTOP1 only in the presence of Al (Supplementary Fig. S5c).

**Figure 3:**
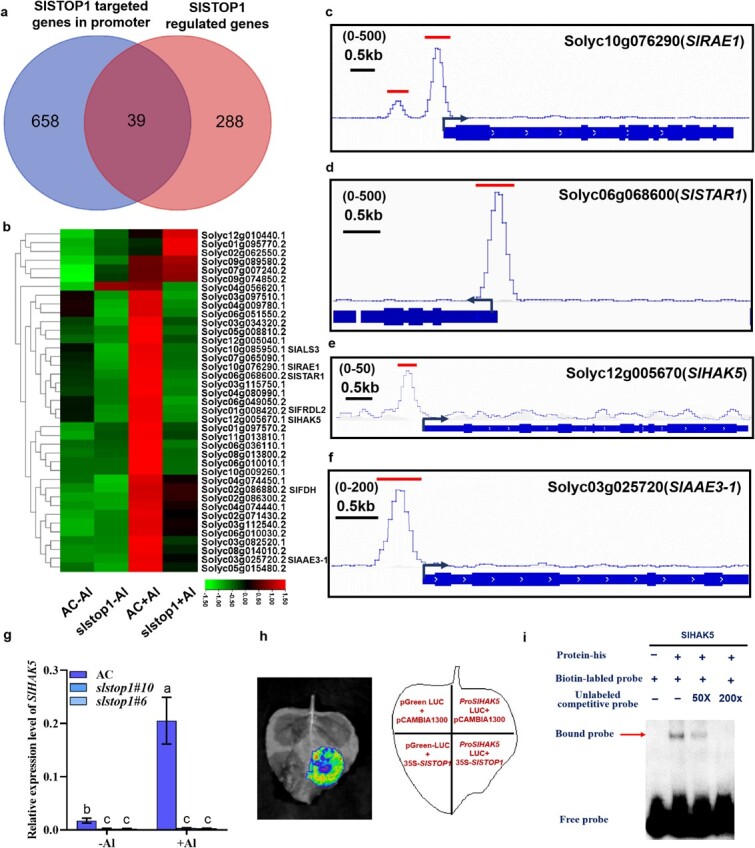
SlSTOP1 binding and its direct regulation on Al-regulated gene clusters. (**a**) Venn diagram shows overlap of SlSTOP1-targeted genes in promoter and SlSTOP1-regulated genes. (**b**) Heat map of expression level of 39 integrated genes in (**a**). (**c–f**) The IGV browser exhibits the binding of SlSTOP1 to four selected genes, namely *SlRAE1* (**c**), *SlSTAR1* (**d**), *SlHAK5* (**e**), and *SlAAE3–1* (**f**). (**g**) *SlHAK5* expression in AC and both *Slstop1* mutants without or with Al treatment for 6 h. *ACTIN* is used as an internal control throughout all experiments conducted herein. Data showed means ± SD. Different letters indicate significant differences among treatments by one-way ANOVA at the *P* ≤ 0.05 level. (**h**) Transient transcriptional activity assays confirm the binding of SlSTOP1 protein to the promoter region of *SlHAK5*. (**i**) EMSA assay validates that SlSTOP1 protein binds to the targeted motif within *SlHAK5* promoter.

To identify direct targets of SlSTOP1 in response to Al stress, we next integrated 327 overlapping DEGs from RNA-seq with promoter-targeted genes from DAP-seq. As a result, 39 genes were identified to be transcriptionally regulated in response to Al stress via the binding of SlSTOP1 to their promoters (Table 1). These SlSTOP1-directly regulated Al-responsive genes can be functionally categorized into transporters, transcription factors, signaling, metabolism, and other genes. Seven of the 39 genes are homologs of known Al tolerance genes, such as *SlALS3*, *SlRAE1*, *SlSTAR1*, *SlFRDL2*, *SlFDH*, *SlAAE3-1*, and *SlPGIP1* (Fig. 3a and b, Table 1), suggesting that these targets may represent the most important Al tolerance genes in tomato. Notably, the selected genes, namely, *SlRAE1*, *SlSTAR1*, *SlHAK5*, and *SlAAE3-1*, were visualized to identify the binding sites for SlSTOP1, which are located near their TSSs (Fig. 3c−f).

**Table 1:** Summary of 39 Al-responsive genes directly targeted by SlSTOP1 binding to the promoters

**Gene ID**	**Gene name**	**Arabidopsis homolog**	**Description**	**Reference**
**Transporter (5)**				
Solyc10g085950.1	*SlALS3*	*AtALS3*	protein ALUMINUM SENSITIVE 3	6
Solyc06g068600.2	*SlSTAR1*	*AtSTAR1*	ABC transporter I family member 17-like	7
Solyc01g008420.2	*SlFRDL2*	*AtMATE*	protein DETOXIFICATION 42-like	23
Solyc12g010440.1	*LePS3*	*AtG3PP5*	putative glycerol-3-phosphate transporter 5	
Solyc12g005670.1	*SlHAK5*	*AtHAK5*	high affinity K+ transporter 5	
**Transcription factor (1)**				
Solyc06g051550.2	*LeFER*	*AtFIT*	bHLH transcriptional regulator	
**Signaling (6)**				
Solyc04g056620.1	*SlPIN5*	*AtPIN5*	auxin efflux carrier component 10	
Solyc03g097510.1	*SlSAUR17*	*AtSAUR17*	hypothetical protein EJD97_006675	
Solyc03g082520.1	*SlSAUR*	*AtSAUR30*	hypothetical protein EJD97_017577	
Solyc04g009780.1	*SlATL71*	*AtATL71*	putative RING-H2 finger protein ATL71	
Solyc12g005040.1	*SlMAPKAPK5*	*AtCPK21*	hypothetical protein EJD97_004450	
Solyc10g076290.1	*SlRAE1*	*AtRAE1*	F-box/LRR-repeat protein 3	19
**Metabolism (11)**				
Solyc02g086300.2	*SlGAT1*		putative glutamine amidotransferase GAT1_2.1	
Solyc03g034320.2	*SlGAT1_2.1*		putative glutamine amidotransferase GAT1_2.1	
Solyc03g115750.1	*SlGT11*		probable xyloglucan galactosyltransferase GT11	
Solyc09g074850.2	*SlGST*		glutathione S-transferase PARB	
Solyc09g089580.2	\	\	1-aminocyclopropane-1-carboxylate oxidase	
Solyc05g008810.2	*SlLPP2L*		lipid phosphate phosphatase 2-like	
Solyc03g112540.2	\	\	short-chain dehydrogenase TIC 32, chloroplastic	
Solyc02g086880.2	*SlFDH*		formate dehydrogenase	16
Solyc03g025720.2	*SlAAE3–1*		oxalate—CoA ligase	15
Solyc11g013810.1	*SlNIA*	*AtNIA1*	nitrate reductase [NADH]	
Solyc07g007240.2	\	\	metallocarboxypeptidase inhibitor-like	
**Others (16)**				
Solyc07g065090.1	*SlPGIP1*	*AtPGIP1*	polygalacturonase inhibitor protein precursor	26
Solyc01g095770.2	*SlCNGC1*	*AtCNGC1*	hypothetical protein EJD97_012638	
Solyc04g080990.1	*SlSLAC1*	*AtSLAH1*	SLAH4-like	
Solyc02g062550.2	\	*AtBCS1*	protein HYPER-SENSITIVITY-RELATED 4	
Solyc06g049050.2	*SlEXPA8*	*AtEXP1*	expansin precursor	
Solyc01g097570.2	\	\	dachshund homolog 1	
Solyc06g036110.1	\	\	uncharacterized protein LOC101263030	
Solyc08g013800.2	\	\	putative F-box/LRR-repeat protein 23	
Solyc06g010010.1	*SlMLOl3*	*AtMLO3*	MLO-LIKE PROTEIN	
Solyc10g009260.1	\	\	hypothetical protein EJD97_018782	
Solyc04g074450.1	*SlEXO*	*AtEXO*	protein EXORDIUM	
Solyc04g074440.1	*SlEXOL*	*AtEXO*	protein EXORDIUM-like	
Solyc02g071430.2	\	\	protein SRG1-like	
Solyc06g010030.2	*SlMLO*	*AtMLO6*	MLO-like protein 6	
Solyc08g014010.2	\	\	uncharacterized protein LOC101249108	
Solyc05g015480.2	\	\	uncharacterized protein LOC101244665	

To provide additional evidence that the transcriptional regulation of these targets requires SlSTOP1, we selected *SlHAK5* expression as an example. While the expression of *SlHAK5* was strongly induced by Al stress in AC plants, it was strongly repressed in the *Slstop1* mutants regardless of Al treatment (Fig. 3g). We performed transient transcriptional activity assays in *Nicotiana benthamiana* leaves and electrophoretic mobility shift assays (EMSAs) to provide *in vivo* and *in vitro* evidence of SlSTOP1 binding to the promoter of *SlHAK5,* respectively. We found that luciferase activity was strongly induced when a reporter vector harboring a 2-kb promoter region upstream of the *SlHAK5* TSS was co-expressed with an effector vector carrying the full-length CDS of *SlSTOP1* (Fig. 3h). Similarly, EMSA experiments revealed that the recombinant SlSTOP1 protein interacted with the targeted promoter regions of *SlHAK5* and that the binding efficiency decreased with increasing amounts of unlabeled competitive probes (Fig. 3i). Collectively, these results showed that SlSTOP1 is required for the transcriptional activation of *SlHAK5* expression.

### The role of SlHAK5 in Al tolerance is independent of K deficiency

Al-induced disorders of mineral nutrients have been reported to be associated with Al toxicity in tomato [27]. Moreover, it has been demonstrated that Al inhibits K^+^, Ca^2+^, and Mg^2+^ transport across the plasma membrane of root cells [28]. Here, we also observed that the K content in the root tips (0–1 cm) decreased with increasing Al concentration (Supplementary Fig. S6), which indicated that Al interferes with K uptake, as previously reported. *SlHAK5*, an ortholog of *AtHAK5* that encodes a high-affinity potassium transporter in *Arabidopsis* [29, 30], was identified as a direct target of SlSTOP1. Since evidence regarding the role of a gene involved in the role of K nutrition in Al tolerance is lacking, we were interested in *SlHAK5*. To this end, we first examined the possible role of SlHAK5 in K nutrition. We investigated the subcellular localization of SlHAK5 by introducing the *35S::SlHAK5::GFP* construct into *N. benthamiana* leaves. The colocalization of the GFP signal with the red fluorescence of the plasma membrane marker AtNIP1;2 suggested that SlHAK5 is a plasma membrane-localized protein (Supplementary Fig. S7a). We then determined the role of *SlHAK5* in terms of K deficiency by developing two *SlHAK5* KO lines by CRISPR/Cas9 technology (Supplementary Fig. S8). Compared with AC plants, both *Slhak5* mutants presented an intensified response to K deficiency, manifested as more pronounced chlorosis and stunted plant growth (Supplementary Fig. S7b−e). Additionally, the K content was much lower in both *Slhak5* mutants than in AC plants under both K-sufficient and K-deficient conditions (Supplementary Fig. S7f), confirming the crucial role of *SlHAK5* in tomato K nutrition [31].

**Figure 4:**
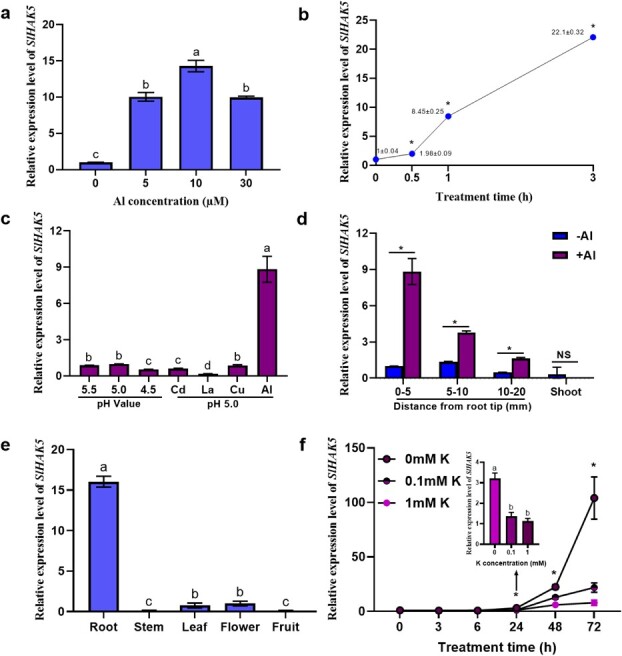
The expression pattern of *SlHAK5*. (**a**) Al dose–response of *SlHAK5* expression. Seedlings of AC with 3–4 cm length of primary root were treated with 0, 5, 10, 20, and 30 μM Al for 6 h. (**b**) Time-course expression of *SlHAK5*. The expression level of *SlHAK5* treated with 5 μM Al for durations of 0, 0.5, 1, and 3 h, respectively. (**c**) The specificity of *SlHAK5* expression. Seedlings of AC with 3–4 cm length of primary root were subjected to different treatments. (**d**) Relative expression levels of *SlHAK5* in root tips (ranging from 0–5, 5–10, and 10–20 mm) and shoot without or with 5 μM Al treatment for 6 h. (**e**) Tissue-specific expression patterns of *SlHAK5* across various tissues including root, stem, leaf, flower, and fruit. (**f**) The effect on *SlHAK5* expression level upon supplementation with 0, 0.1, and 1 mM K supplement for 0, 3, 6, 24, 48, and 72 h. *ACTIN* used as an internal control. All experiments were performed at least two times independently and similar results were observed. Data are means ± SD (*n* = 3 for technical replicates). Different letters and * indicate significant differences among treatments by one-way ANOVA at the *P* ≤ 0.05 level.

Considering that the dramatic induction of *SlHAK5* expression by Al stress relies on SlSTOP1 (Fig. 3g), we next focused on the role of SlHAK5 in Al tolerance by investigating its expression pattern. In a dosing experiment, the expression level of *SlHAK5* increased with increasing Al concentration, peaked under 10 μM Al, and then slightly decreased under higher Al concentrations (Fig. 4a). In a time-course experiment, the expression of *SlHAK5* was induced quickly by 0.5 h of Al exposure and increased further with prolonged exposure time (Fig. 4b). To examine the specificity of *SlHAK5* expression, we analyzed *SlHAK5* expression in response to various pH values and different metals. *SlHAK5* expression was only induced by Al but not by the other metal treatments, and lower pH (4.5), Cd, or La treatment even slightly inhibited *SlHAK5* expression, indicating that the expression of *SlHAK5* was specific to Al (Fig. 4c). The Al-induced expression of *SlHAK5* was confined to the roots and absent in the shoots. Moreover, the apex of the apical roots (0–5 mm) presented greater induction of expression than did the basal roots under Al stress (Fig. 4d). The tissue-specific expression of *SlHAK5* revealed that it was expressed mainly in the roots but was weakly expressed in other organs, including the stem, leaf, flower, and fruit (Fig. 4e). These results indicate a potential role for SlHAK5 in regulating Al-induced responses in tomato.

A previous study reported that Al inhibits K^+^ uptake by reducing the activity of AKT1, a K^+^ channel [32]. Moreover, HAK5 functions as another high-affinity K^+^ transporter, and its expression can be induced by low external K^+^ concentrations in *Arabidopsis* [29]. Therefore, we wondered whether the Al-induced expression of *SlHAK5* resulted from Al-induced K deficiency. When AC plants were subjected to different K concentrations (0, 0.1, and 1 mM), *SlHAK5* expression was induced by K deficiency after 24 h, which was much slower than its rapid induction by Al within just 0.5 h (Fig. 4f). After 3 days of K deficiency, the shoots of K-deficient plants were much smaller than those of K-sufficient plants. Moreover, while K deficiency resulted in a remarkable reduction in both the root and shoot K levels compared with those under K sufficiency, exposure to Al did not significantly affect the K content, irrespective of the K status (Supplementary Fig. S9). These results suggest that the Al-induced expression of *SlHAK5* does not directly result from Al-induced K deficiency.

### SlHAK5 is positively involved in Al-induced citrate secretion for Al tolerance

**Figure 5:**
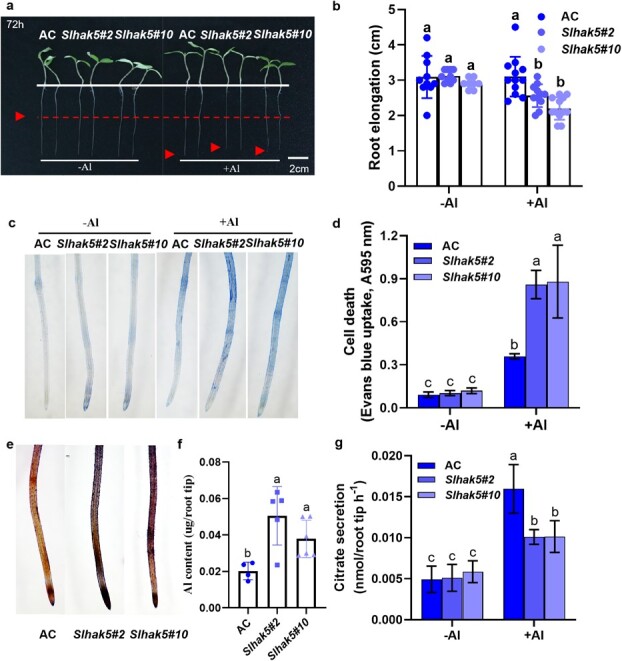
*Slhak5* mutants showed increased sensitivity to Al stress. Phenotype of representative seedlings (**a**) and primary root elongation (**b**) of AC and both *Slhak5* mutants. Roots with ~3–4 cm in length were subjected to Al treatment, and their length was measured before and after Al treatment. (**c**) Evans blue staining. After treatment, roots of AC and two *Slhak5* KO lines were stained with Evans blue staining solution, washed, and photographed by microscope. (**d**) Determination of the cell death in roots. Evans blue staining roots were extracted with 1% SDS and detected at A595 nm. (**e**) Hematoxylin staining revealed Al content in roots of AC and both *Slhak5* mutants under Al treatment. Al-treated roots of AC and *Slhak5* mutants were stained with hematoxylin, washed, and photographed afterwards. Bar = 0.5 mm. (**f**) The total Al content in 0- to 1-cm root tips of AC and two *Slhak5* KO lines. After treatment, root apices were collected for extraction of Al and analyzed by ICP–MS. (**g**) Measurement of citrate in solution bating the roots of AC and two *Slhak5* KO lines. AC and two *Slhak5* KO lines were subjected to 0 or 5 μM Al for 72 h, then root tips (0–1 cm) were cut, washed briefly, and placed in a 0.5 mM CaCl_2_ solution to collect the root exudates for 12 h. Citrate was determined via an enzymatic method. Data are means ± SD (*n* = 12). Different letters indicate significant differences among treatments by one-way ANOVA at the *P* ≤ 0.05 level.

To examine the role of *SlHAK5* in Al tolerance, AC plants and two *Slhak5* KO lines were exposed to 0 and 5 μM Al solutions for 72 h to measure root elongation. While 5 μM Al had no effect on the growth of the roots of AC plants, it significantly inhibited the elongation of the roots of the two *Slhak5-*KO lines (Fig. 5a and b). Moreover, this difference in root elongation between AC and the two *Slhak5* KO lines became more evident at higher Al concentrations (Supplementary Fig. S10a). Accordingly, Evans blue staining also revealed more severe cell death in the two *Slhak5* KO lines than in the AC plants under Al treatment (Fig. 5c and d). Additionally, the Al content in the two *Slhak5* KO lines was determined by staining with the Al indicator dye hematoxylin or via ICP–MS analysis, which revealed a greater content than that in the AC root tips (Fig. 5e and f). These results suggest that *SlHAK5*-mediated Al tolerance is associated with external Al exclusion. Since organic acid anion secretion has been recognized as the most important Al exclusion mechanism [2], we subsequently measured the exudation of citrate, malate, and oxalate in response to Al stress. Among the three organic acid anions, oxalate was secreted the most, followed by citrate, and malate secretion was almost undetectable. While the secretion of oxalate in AC roots was constitutive, the secretion of citrate was strongly induced by Al stress (Supplementary Fig. S11). Therefore, it appears that Al-induced citrate secretion is important for acquired resistance in tomato, whereas oxalate secretion may contribute to the basal resistance of tomato to Al toxicity. Intriguingly, the Al-induced secretion of citrate was significantly lower in the *Slhak5* KO lines than in the AC plants (Fig. 5g). To further examine whether K^+^ nutrition is involved in Al tolerance, we examined the effects of different levels of K nutrition on root elongation under high concentrations of Al (15 μM). In the absence of Al, a high K concentration (20 mM) had no significant effect on root elongation. However, in the presence of 15 μM Al, root elongation was considerably greater at high K concentrations than at low K concentrations (1 mM) in both AC and the two *Slhak5* KO lines (Supplementary Fig. 10b and c). Concomitantly, the amount of citrate secreted was much greater at high K concentrations than at low K (1 mM) (Supplementary Fig. 10d). These findings suggest the positive involvement of SlHAK5 in Al-induced citrate secretion, which contributes to reducing the internal accumulation of Al in root tips and improving tolerance to Al stress.

## Discussion

STOP1 acts as a central hub to control Al tolerance by regulating a variety of downstream genes, which are conserved in various plant species [33, 34]. Like its homologs AtSTOP1 in *Arabidopsis* and OsART1 in rice [9, 12], a loss-of-function mutation in SlSTOP1 resulted in hypersensitivity to Al stress (Fig. 1), highlighting that SlSTOP1 serves as a master regulator of Al tolerance in tomato. Previous studies have identified the targets of AtSTOP1 in Arabidopsis and ART1 in rice [11, 12]. However, the specific targets of STOP1 proteins differ among plant species, contributing to the diversity of STOP1-regulated Al tolerance mechanisms in plants. For example, while AtSTOP1 regulates the expression of genes involved not only in Al tolerance but also in low pH tolerance, rice ART1 only regulates Al tolerance genes. Here, we provide comprehensive and novel insights into how SlSTOP1 mediates Al tolerance in tomato by identifying the direct targets of Al-responsive genes. A screen of SlSTOP1-targeted genes very likely successfully identified the majority of critical genes involved in the response to Al stress. For example, we observed high-affinity promoter binding of SlSTOP1 to multiple promoter regions of *SlALS3*, *SlSTAR1*, *SlHAK5*, and *SlAAE3-1* (Fig. 3a−f), which is essential for its transcriptional induction under Al stress. Importantly, both *SlALS3* and *SlSTAR1* presented high sequence similarity to their *Arabidopsis* homologs *AtALS3* and *AtSTAR1*, respectively, indicating that these two genes play similar roles in terms of Al tolerance in tomato. A previous study demonstrated the involvement of *SlAAE3-1*, a target of SlSTOP1, in Al tolerance in tomato [15]. Consistent with our previous report [16], we found that SlSTOP1 directly regulated *SlFDH* expression by targeting its promoter. However, we also found that SlSTOP1 directly targeted *SlSTOP2L* but did not regulate its expression (Fig. 2). These findings support the assumption that the regulatory systems of STOP1 are variable, although this protein is functionally conserved among plant species.

Despite being a master regulator of low pH and Al tolerance, AtSTOP1 in Arabidopsis serves as a pleiotropic transcription factor that participates in multiple stress responses, such as low phosphate, low potassium, drought, and salt stress [33]. Under low pH conditions, AtSTOP1 is involved in increasing rhizosphere pH by regulating *NRT1.1* expression to facilitate nitrate uptake in Arabidopsis [35]. In the present study, the identification of genome-wide binding targets of the SlSTOP1 protein revealed that its targets are involved in multiple pathways, with the ‘response to temperature stimulus’ pathway being the most enriched in the GO enrichment analysis of all the targeted peaks (Fig. 2a and b), suggesting the potential involvement of SlSTOP1 in the response to heat stress, which needs further investigation.

The transcription of *SlSTOP1* was unaffected by Al stress, while the accumulation of SlSTOP1 proteins was observed (Supplementary Fig. S1). These findings are consistent with those of previous reports on *Arabidopsis* AtSTOP1, which revealed that posttranscriptional and posttranslational modifications are involved in regulating STOP1 protein accumulation under Al stress. For example, HPR1 (hyperrecombination protein 1), a component of the THO/TREX complex, facilitates the export of AtSTOP1 mRNA from the nucleus to the cytoplasm [36]. Moreover, AtSTOP1 targeted *RAE1* (*Regulation of AtALMT1 Expression 1*) to upregulate its expression, whereas RAE1 reciprocally promoted AtSTOP1 degradation via the ubiquitin 26S proteasome pathway, forming a feedback loop in response to Al stress [19]. In addition, SUMOylation, the phosphatidylinositol-specific phospholipase C (PI–PLC) pathway, and H_2_O_2_-mediated phosphorylation and oxidative modification have also been implicated in promoting the nuclear accumulation of STOP1 at the posttranslational level [11, 37–40]. Proper STOP1 protein accumulation is clearly vital for the transcriptional activation of downstream target genes. In this study, we identified *SlRAE1* as one of the direct targets of SlSTOP1, suggesting that the STOP1-RAE1 module may be conserved among different plant species to prevent the overaccumulation of STOP1 proteins. However, further exploration is needed to investigate other potential processes involved in the posttranscriptional modification of SlSTOP1.

Transcription factors (TFs) regulate gene expression by interacting with the *cis*-acting elements present in the promoters of target genes. For the rice ART1 protein, an ortholog of Arabidopsis AtSTOP1, a consensus sequence (GGNVS) was characterized as the binding motif [41]. However, this reported motif is short and highly degenerate, limiting its utility in identifying genes targeted by STOP1 or ART1 orthologs. A longer sequence of TAAGGGGAGGGC in the *AtALMT1* promoter was subsequently identified to be directly targeted by AtSTOP1 to regulate *AtALMT1* expression [42]. Similarly, AtSTOP1 harbors the GGGGAGGGCTTAACT sequence of the *AtALMT1*, *AtSTOP2*, and *AtGDH1/2* promoters to increase their transcription [11]. Additionally, AtSTOP1 has been reported to bind the *NRT1.1* promoter at the AAGCTACCCGAGATATCACCTTCTTTTGGTAT sequence [43]. Recently, several studies have focused on the STOP1 binding motif CCTTCCTCG, which is shared by the *RAE1*, *CIPK23*, and *LKS1* promoters [19, 44, 45]. Here, a core sequence of 5′-CCTTCCCCAGATAA-3′ was enriched in all SlSTOP1-targeted peaks (Fig. 2g), which is similar to the SlSTOP1-targeted *SlFDH* promoter sequence TCCTTCCCCAAAAAA reported in our previous study [16]. On the basis of this information, we conclude that STOP1 can notably bind multiple motifs to regulate the expression of different target genes.

It has been demonstrated that the uptake of Al impedes the absorption of K by reducing the open probability and activation kinetics of KAT1 [32]. However, how K^+^ uptake affects Al tolerance remains unknown. Here, we identified *SlHAK5* as a novel Al-responsive gene targeted directly by SlSTOP1. This conclusion is based on the following pieces of evidence. First, *SlHAK5* expression was specifically induced in roots by Al stress, a process that was not related to Al-induced K deficiency (Fig. 4). Although *HAK5* expression is typically induced by K deficiency [29], our results revealed a rapid induction of *SlHAK5* under Al stress compared with the longer time required for that under K deficiency (Fig. 4). Moreover, regardless of their K status, we observed no significant effects on either the root or shoot K content under Al stress (Supplementary Fig. S9). Second, the *Slhak5* KO lines were more sensitive to Al stress than the AC plants were, which was correlated with a decrease in Al-induced citrate secretion in the two *Slhak5* KO lines (Fig. 5). Third, extra K supplementation alleviated Al-induced root elongation inhibition, which was accompanied by greater citrate secretion from the root apex (Supplementary Fig. S10b–d). Considering that K functions as an enzyme cofactor and plays roles in osmoregulation, plasma membrane potential maintenance, and ATP synthesis [29], the induction of *SlHAK5* under Al stress may be favorable for maintaining these physiological and biochemical processes, thereby improving citrate secretion. In agreement with our viewpoint, Mg transporter OsMGT1-mediated Mg uptake has been reported to be required for preventing the cellular dysfunction caused by Al toxicity [46]. However, Mg-mediated Al tolerance is not related to citrate secretion, possibly because different strategies have evolved for resisting Al toxicity in rice and tomato. While tomato relies on citrate secretion to exclude Al, citrate secretion plays a minor role in rice Al resistance [26].

In *Arabidopsis*, a well-characterized regulatory pathway that enhances K^+^ uptake under low-K conditions is the phosphorylation of AtHAK5 by the calcineurin B-like protein 1 (CBL1)-CIPK23 (CBL-INTERACTING PROTEIN KINASE23) signaling module [47]. Furthermore, several TFs that positively or negatively regulate *AtHAK5* expression have been reported [48]. Recently, Feng et al. (2021) reported that MYB77 positively regulates HAK5 expression under low-K conditions in Arabidopsis [49]. However, the expression of AtHAK5 was only moderately repressed in *myb77* mutants under low-K conditions, suggesting that other regulators play critical roles in regulating *AtHAK5* expression. Additionally, AtSTOP1 has been identified as a direct regulator of *CIPK23* transcription, thereby controlling multiple ion transporters, including AtHAK5, to confer salt, drought, and low-K tolerance [45, 50]. Therefore, our understanding of the specific transcription factors responsible for regulating *HAK5* expression at the transcriptional level remains limited. Here, we demonstrated that SlSTOP1 directly regulates *SlHAK5* expression in both the presence and absence of Al stress, and notably, the induction of expression was almost completely abolished in the *Slstop1* mutants (Fig. 3), highlighting a distinct transcriptional regulatory mechanism for *SlHAK5* transcription in tomato. The finding that the SlSTOP1-SlHAK5 module directly affects K^+^ nutrition may provide new insight into the regulation of plant K^+^ nutrition.

**Figure 6:**
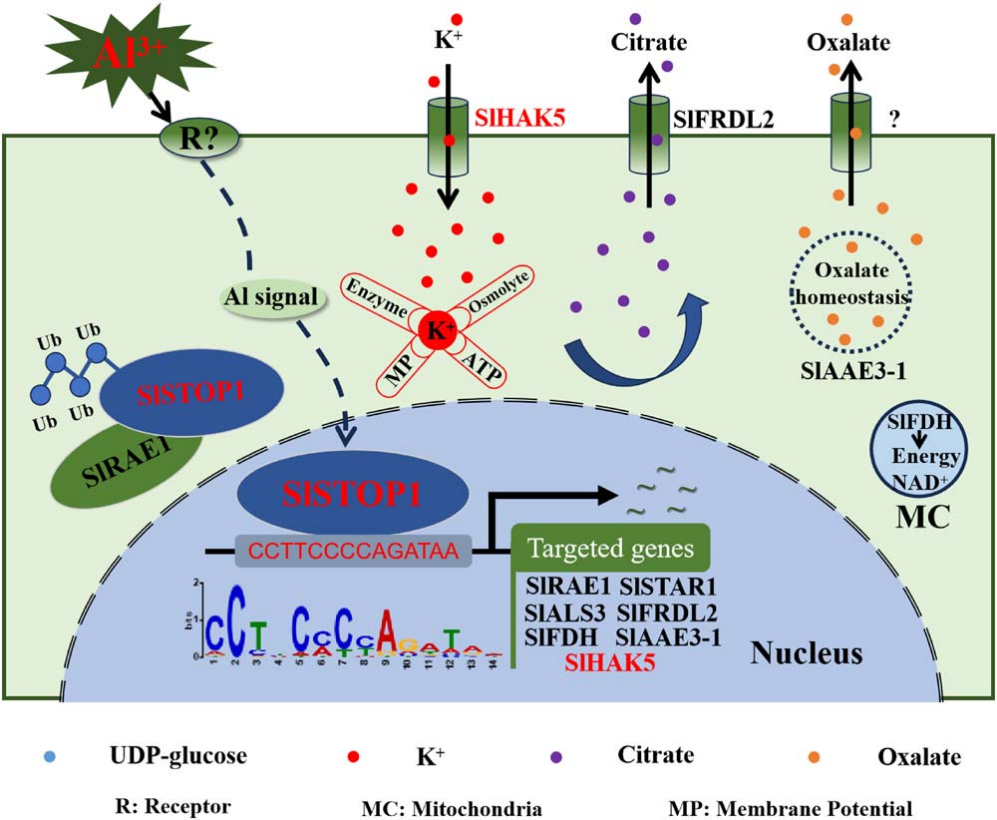
A proposed model for possible roles of SlSTOP1-targeted genes in response to Al stress in tomato. When suffering from Al stress, SlSTOP1 protein accumulated in the nucleus, which directly activates a series of targeted genes, thereby helping plants in resisting Al toxicity. Notably, SlHAK5 as one of SlSTOP1-targets is positively implicated in Al tolerance by improving citrate secretion under Al stress, possibly by maintaining a variety of physiological and biochemical processes.

In summary, we successfully identified multiple SlSTOP1-targeted genes, including *SlRAE1*, *SlSTAR1*, *SlALS3*, *SlFRDL2*, *SlFDH*, *SlAAE3-1*, and *SlHAK5*, via integrative analysis of DAP-seq and RNA-seq data. Moreover, we identified a practical and convincing SlSTOP1-binding motif through systemic enrichment analysis of SlSTOP1 targets of Al-responsive genes. These SlSTOP1-targeted genes play crucial roles in diverse pathways that collectively manage Al toxicity (Fig. 6). Notably, the functional characterization of a high-affinity K^+^ transporter gene, *SlHAK5,* revealed its involvement in conferring Al tolerance by facilitating Al-induced citrate secretion in tomato. These results pave the way for the systematic elucidation of the molecular mechanisms of Al tolerance mechanisms in an important vegetable crop.

## Materials and methods

### Plant materials and treatment conditions

The tomato (*S. lycopersicum*) cultivar Ailsa Craig (AC) was used for this study, and all transgenic plants were generated in the AC background. Similar CRISPR/Cas9 gene-edited *Slstop1* and *Slhak5* mutant lines were developed by GIOGLE Gene Tech (Hangzhou, China). For the generation of *35S::SlSTOP1::GFP* and *ProSlSTOP1::GUS* transgenic plants, the full-length coding sequence of *SlSTOP1* without the stop codon and the 2-kb promoter region of *SlSTOP1* were amplified and cloned and inserted into the pCAMBIA1300-GFP vector and pCAMBIA1300-NosP::HPT-GUS Plus, respectively. These constructs were subsequently separately transformed into AC plants via hygromycin selection to obtain homozygous transgenic plants. Two *35S::SlSTOP1::GFP* homozygous transgenic lines and three *ProSlSTOP1::GUS* homozygous transgenic lines were used for further analysis.

Sterilized seeds were germinated in petri dishes containing one-fifth-strength Hoagland solution with 1% agar. Uniformly grown seedlings were transferred to treatment solutions supplemented with or without Al (one-fifth Hoagland with 10 μM NH_4_H_2_PO_4_, pH 5.0) when the primary roots were ~3 to 4 cm in length. To observe GUS or GFP activity, the seedlings were treated with or without 5 μM Al for 6 h. To assess Al tolerance, *Slstop1* or *Slhak5* mutant seedlings were treated with or without 5 μM Al for 48 or 72 h. To investigate the effect of Al treatment on root elongation in the *Slhak5* mutant, a time-course experiment was conducted in which 5 μM Al was used for durations of 6, 12, 24, 36, and 48 h. Subsequently, a dosing experiment was carried out in which 0, 2.5, 5, and 10 μM Al were used for a duration of 6 h. To analyze the phenotype of the *Slhak5* mutant under K deficiency, the seedlings were treated with or without K supplementation for 2 weeks. An Al concentration gradient of 0, 5, 10, and 15 μM was applied to the AC and two *Slhak5* KO lines for 72 h, and then 15 μM Al supplemented with K was selected for further treatment.

### GUS and GFP observation

Different tissues and roots treated with or without Al were subjected to GUS activity determination via histochemical staining. Three-month-old *ProSlSTOP1::GUS* transgenic plants were used to analyze the *SlSTOP1* expression pattern via GUS staining. All the tissues except the roots were transferred into 70% ethanol ~2 to 3 times until complete decolorization was achieved after incubation in GUS stain at 37°C for 1 h or overnight. The stained tissues were either directly observed via microscopy or observed after decolorization. To visualize the GFP fluorescence emitted from the SlSTOP1-GFP fusion protein, the roots of *35S::SlSTOP1::GFP* transgenic plants were observed for GFP fluorescence via fluorescence microscopy after 0 or 5 μM Al treatment. The fluorescence intensity was subsequently quantified with ImageJ. To determine the subcellular localization of *SlHAK5* through GFP fluorescence, a protocol similar to that described by Zhu et al. (2022) was employed, where AtNIP1;2-RFP served as a marker for the plasma membrane [51].

### Phenotype analysis under Al stress

The measurement of root elongation involved assessing the root length before and after Al treatment. The phenotype was subsequently photographed with a digital camera after Al treatment. For Evans blue staining, the roots were immersed in 0.25% Evans blue staining solution for 30 min and observed under a digital camera after being washed with ddH_2_O. The stained roots were subsequently washed with a 1% SDS solution, and the resulting extracts were quantified at 595 nm. For Al content measurement, hematoxylin staining and total Al quantification via ICP–OES were performed according to a previous report [24]. Approximately 3- to 4-cm-long primary roots were treated with 0 or 5 μM Al, and then, the tips (0–1 cm) were cut, washed briefly, and placed in a 0.5 mM CaCl_2_ solution to collect the root exudates for 12 h. Citrate, oxalate, and malate contents were determined via an enzymatic method according to previous methods described by Delhaize et al. (1993) [52].

### DAP-seq analysis

The DAP-seq assay was conducted as described previously with minor modifications [53]. Briefly, genomic DNA was isolated from tomato leaves and then fragmented to ~200 bp in length using a Covaris M220 (Woburn, MA, USA), and the fragmented DNA was then purified via MICH DNA Clean Beads (Cat# NGS0201, Bluescape Hebei Biotech Co., Ltd). To construct the DAP-seq genomic DNA library and express the Halo-SlSTOP1 fusion protein, we utilized the MICH TLX DNA-Seq Kit (Cat# NGS0602, Bluescape Hebei Biotech Co., Ltd) and the TNT SP6 Coupled Wheat Germ Extraction System (Promega), respectively. The DAP-seq binding assay was performed, and the resulting bound DNA fragments were sequenced on an Illumina NovaSeq platform. Negative control mock DAP-seq libraries were prepared without adding protein to the beads. The raw data were filtered to obtain clean data, aligned to the tomato genome sequence via BWA-MEM, and then filtered for reads containing >MAPQ30 via SAM tools. MACS2 call peak and Homer software were used for merging the peaks from two biological replicates with a significance level of Q < 0.05. To identify motifs and annotate bound peaks, MEME-ChIP software and ChIP seeker software were employed separately.

### Quantitative real-time PCR and RNA-seq analysis

To analyze *SlHAK5* gene expression, the treatment conditions for the gene expression pattern were consistent with those previously described by He et al. (2023) [16], and the expression level of *SlHAK5* was determined with 0, 0.1, or 1 mM K supplementation for 0, 3, 6, 24, 48, and 72 h. The process of total RNA extraction involved the use of an RNAprep Pure Plant Kit, while reverse transcription was performed with PrimeScript RT Master Mix. The qPCR assay was subsequently performed on a LightCycler 480 device with SYBR Green serving as the detection dye. The primer information is shown in Supplementary Table S7. RNA-seq was performed on the Illumina HiSeq platform with three biological replicates of each treatment, as described in a previous study [54].

### Dual-luciferase transcriptional activity assay

The 2-kb region of the *SlHAK5* promoter was amplified via specific primers (Table S7) and subsequently cloned and inserted into pGreenII 0800-LUC to generate a LUC reporter, and the 35S::SlSTOP1::GFP construct was used as an effector. To measure LUC activity, a pair of reporters and effectors was transformed into *N. benthamiana* leaves according to the established protocol described by He et al. (2023) [16].

### Electrophoretic mobility shift assay

The recombinant SlSTOP1 protein was induced and purified as described in our previous study [16]. For the EMSA, the binding motif of the SlSTOP1-targeted *SlHAK5* promoter identified via DAP-seq was synthesized with or without a biotin label as a probe.

## Supplementary Material

Web_Material_uhae282

## Data Availability

The raw data of DAP-seq and RNA-seq were uploaded into NCBI under accession number PRJNA1050863 and PRJNA1052400, respectively.
