# Supplementary material for: SlSTOP1-regulated SlHAK5 expression confers Al tolerance in tomato by facilitating citrate secretion from roots
Source: Hortic Res. 2024 Oct 2;11(11):uhae282. doi: 10.1093/hr/uhae282 (PMC11561044; doi:10.1093/hr/uhae282)
Supplement: Web_Material_uhae282 [file web_material_uhae282.docx]

**Figure S1.** *SlSTOP1* expression pattern analysis. **a** Tissue-specific expression analysis of *SlSTOP1* in *ProSlSTOP1::GUS* reporter lines. **b** Al treatment had not effects on *SlSTOP1* expression. *ProSlSTOP1::GUS* reporter lines with primary root length of about 3-4 cm were subjected to 0 or 5 μM Al for 6 h. **c** RT-qPCR analysis of SlSTOP1 expression at the flowering stage (three-month-old). **d** the GUS staining in leaves of 3 independent transgenic GUS reporter lines **e** SlSTOP1 proteins were accumulated in tomato root tip under Al stress. The *35S::SlSTOP1::GFP* transgenic reporter lines with primary root length of 3−4 cm were subjected to 0 or 5 μM Al for 6 h. GFP fluorescence was observed by fluorescence microscope. And the punctiform GFP fluorescence represents the nucleus location of SlSTOP1 **f** Quantification of GFP fluorescence from recombinant SlSTOP1-GFP protein in root tips. Letters indicated significant difference. Scale bars in (a), (b) and (d) are 1 cm, and in (c) are 1 mm, respectively.


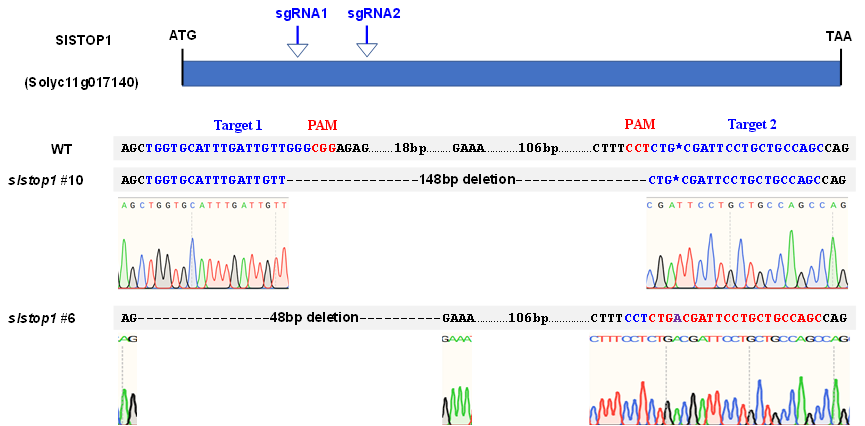


**Figure S2.** Schematic diagrams of *Slstop1* mutants. CRISPR/Cas9-induced gene editing system was used to produce knockout mutants of *SlSTOP1* (Solyc11g017140). A pair of sgRNA target sites in SlSTOP1 coding sequence (251−273 bp for sgRNA1, 410−432 bp for sgRNA2), related PAM sites and CRISPR edited sites are represented in blue, red, and black, respectively. In *Slstop1*#10, a deletion of 148 bp was observed between two targeted sites; in *Slstop1*#6, there was a 48-bp deletion at 1^st^ targeted site and a 1-bp insertion at 2^nd^ targeted sites, respectively.


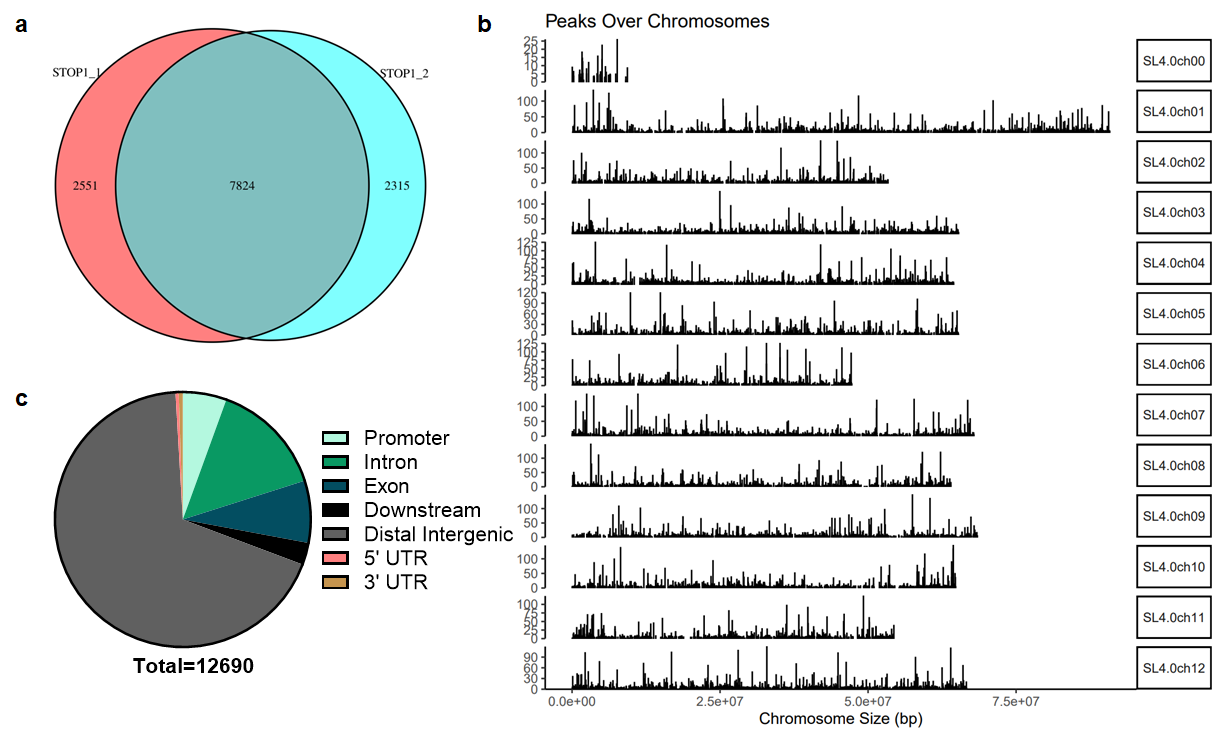


**Figure S3.** Peak distribution of SlSTOP1 targets in tomato genome. **a** Overlapping peaks between two biological replicates of DAP-seq using SlSTOP1 protein. **b** The peak distribution among chromosomes. **c** The distribution in gene features.


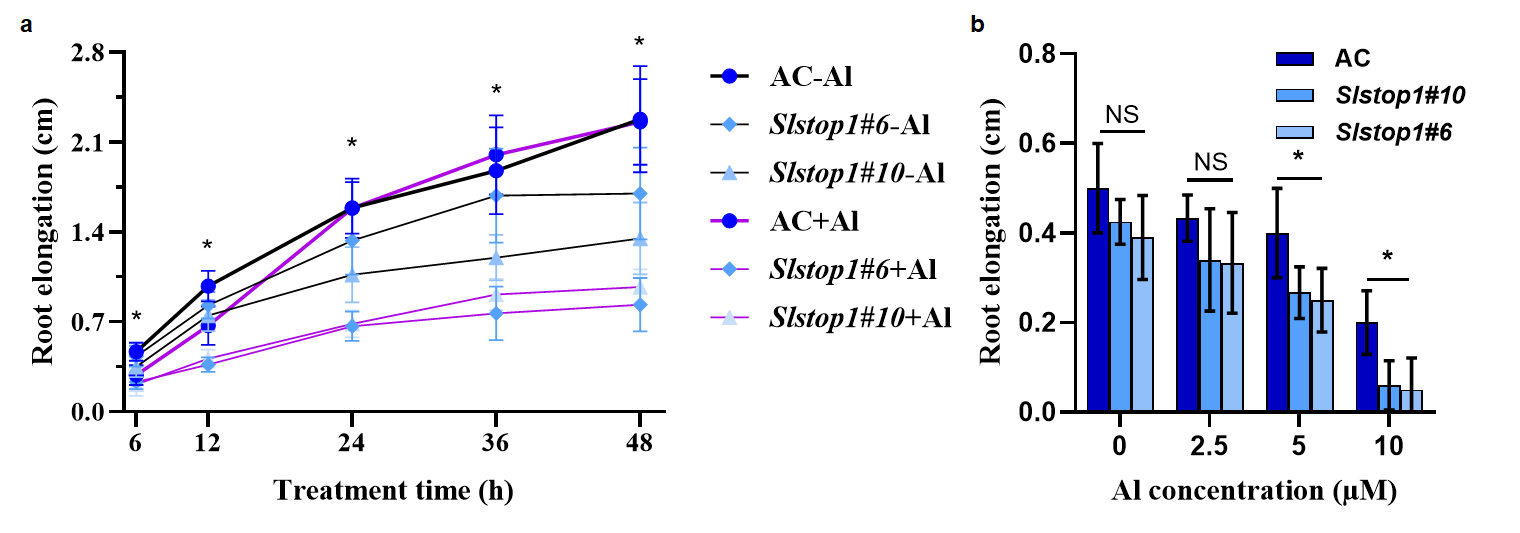


**Figure S4.** Effects of Al treatment on root elongation of *Slstop1* mutants. **a** Root elongation between AC and both *Slstop1* mutants without or with 5 µM Al treatment for durations of 6, 12, 24, 36, 48h, respectively. **b** Root elongation between AC and both *Slstop1* mutants under various concentrations of Al treatment (0, 2.5, 5, 10 µM) for 6h each. Seedlings of AC and *Slstop1* mutants approximately around 3−4 cm in root length seedlings were subjected to either Al treatment or no treatment at all. Root elongation were measured before and after the period of Al treatment. Data are presented as means ± SD. Asterisks indicate significant differences among genotypes based on one-way analysis of ANOVA at a significant level of P≤0.05.


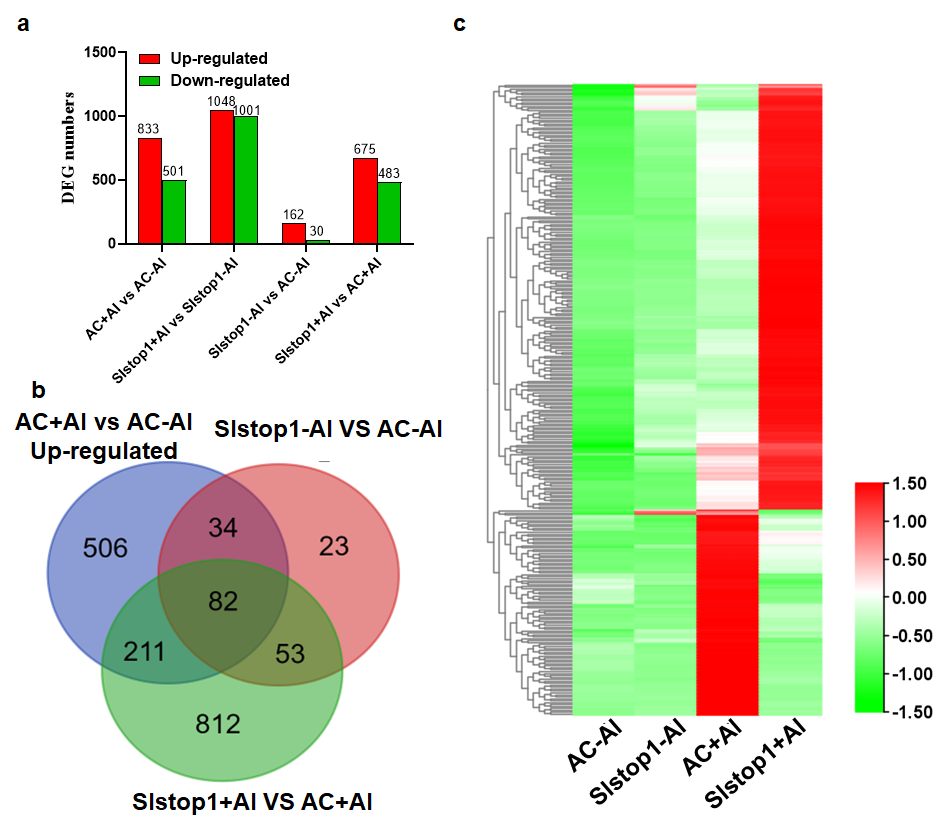


**Figure S5** RNA-seq analysis of SlSTOP1-regulate genes. **a** The number of differentially expressed genes (DEGs) that were up-/down-regulated in various comparisons. **b** Venn analysis illustrates the overlap among “*Slstop1*−Al VS AC−Al”, “*Slstop1*+Al VS AC+Al” and upregulated DEGs induced by Al in AC plants. **c** A heatmap visually displays the overlapped genes from Venn analysis.

**Figure S6.** The K content in root tips (0-1cm) under Al concentration gradients (0, 5, 10, 15 µM Al treatment). “*” indicate significant differences among treatments by one-way ANOVA at the P≤0.05 level.

**Figure S7.** The function of SlHAK5 in K deficiency. **a** Subcellular localization of SlHAK5 in tobacco leaves. *Agrobacterium tumefaciens* carrying recombinant SlHAK5-GFP and plasma membrane marker AtNIP1;2-RFP were co-transformed into *Nicotiana benthamiana* leaves. Subsequently, the green and red fluorescent signals emitted from leaf cells were visualized to determine the subcellular location of SlHAK5. **b−c** The phenotype of K deficiency for two weeks in AC and both *Slhak5* lines. **d−f** The plant height (d), biomass (e) and K content (f) of AC and both *Slhak5* lines under K deficiency. Seedlings with approximately 3−4cm in root length were treated under K deficiency or K sufficiency for two weeks. Means ± SD (n=6) with different letters indicating significant differences among treatments by one-way ANOVA (P≤0.05).


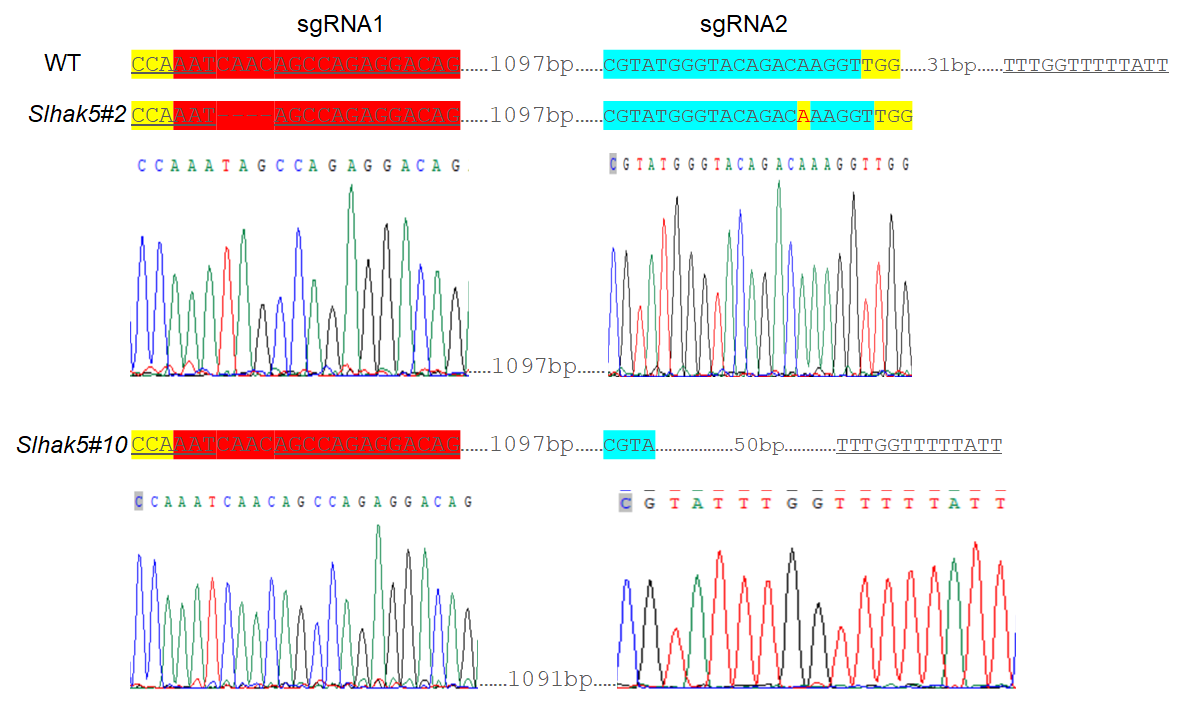


**Figure S8.** Schematic diagrams of *Slhak5* mutants. With the usage of CRISPR/Cas9-induced gene editing system, a pair of sgRNA target sites in *SlHAK5* (Solyc12g005670) coding sequence were highlighted in red and blue, respectively. Short lines indicate base deletions, and red bases represent base insertions. In *Slhak5#2*, a 4-bp deletion occurred in target 1 and a 1-bp insertion occurred in target 2. In *Slhak5#10*, only a deletion of 50 bp was observed in target 2.

**Figure S9.** The effect of K on Al toxicity. **a−b** The phenotype and the relative root elongation of AC plants without or with external K supplementation under 0 and 5 µM Al treatment for 3 d. The root length was measured before and after treatment. **c−d** The K content in roots (c) and shoots (d) of AC plants without or with K supplementation under 0 and 5 µM Al treatment for 3 d. Data are Means ± SD. Bars labeled with ‘NS’ indicate no statistical significance between treatments, while those labeled with different letters indicate significant differences among treatments by one-way ANOVA at the P≤0.05 level

**Figure S10.** The role of SlHAK5-mediated K on Al toxicity. **a** The root elongation AC and two *Slhak5* knockout lines under Al concentration gradient treatments for 72h. The root length was measured before and after Al treatment. **b** The phenotype of AC and two *Slhak5* knockout lines without or with external K supplementation under 0 and 15 µM Al treatment for 3 d. The root length was measured before and after treatment. **c−d** The root elongation (c) and citrate secretion (d) of AC and two *Slhak5* knockout lines without or with K supplementation under 0 and 15 µM Al treatment for 3 d. Data are Means ± SD. Bars labeled with different letters indicate significant differences among treatments by one-way ANOVA at the P≤0.05 level.

**Figure S11.** Organic acid secretion in AC root tips. Seedlings of AC with 3-4 cm length of primary root were treated with 0 or 5 µM Al for 48 h and then root tips (0–1 cm) were cut, washed briefly, and placed in a 0.5 mM CaCl_2_ solution to collect the root exudates for 12 h. Citrate, oxalate and malate was determined via an enzymatic method.
